# Mirror-Touch Synaesthesia Is Not Associated with Heightened Empathy, and Can Occur with Autism

**DOI:** 10.1371/journal.pone.0160543

**Published:** 2016-08-04

**Authors:** Simon Baron-Cohen, Emma Robson, Meng-Chuan Lai, Carrie Allison

**Affiliations:** 1 Autism Research Centre, Department of Psychiatry, University of Cambridge, Cambridge, United Kingdom; 2 Cambridge Lifespan Asperger Syndrome Service (CLASS) Clinic, Cambridgeshire and Peterborough NHS Foundation Trust, Cambridge, United Kingdom; 3 Child and Youth Mental Health Collaborative at the Centre for Addiction and Mental Health and The Hospital for Sick Children, Department of Psychiatry, University of Toronto, Toronto, Canada; 4 Department of Psychiatry, National Taiwan University Hospital and College of Medicine, Taipei, Taiwan; University of Tokyo, JAPAN

## Abstract

Research has linked Mirror-Touch (MT) synaesthesia with enhanced empathy. We test the largest sample of MT synaesthetes to date to examine two claims that have been previously made: that MT synaesthetes (1) have superior empathy; and (2) only ever experience their MT synaesthesia in response to viewing a *person* being touched. Given that autism has been suggested to involve deficits in cognitive empathy, we also test two predictions: that MT synaesthetes should (3) be less likely than general population individuals without MT synaesthesia to have an autism spectrum condition (ASC), if MT is characterized by superior empathy; and (4) have fewer autistic traits. We selected three groups: a pure MT synaesthesia group (*N* = 46), a pure grapheme-colour (GC) synaesthesia group (*N* = 36), and a typical control group without synaesthesia (*N* = 46). Participants took three measures of empathy and one measure of autistic traits. MT synaesthetes did not show enhanced empathy. In addition, 30% of all MT synaesthetes recruited into this study (*N* = 135) reported also having ASC, and MT synaesthetes showed higher autistic trait scores than controls. Finally, some MT experiences were reported in response to viewing objects being touched. Our findings dispute the views that MT synaesthesia is linked with enhanced empathy, is less likely to occur with ASC or elevated autistic traits, and is specific to seeing a person being touched.

## Introduction

Synaesthesia occurs when stimulation in one sensory modality elicits an automatic response in another unstimulated perceptual modality [1]. Examples include pain-inducing colours [2], tones of music evoking colour [3, 4], or grapheme-colour synaesthesia [5]. Synaesthesia research dates back to 1812, which provided an account of an albino music-colour and grapheme-colour synaesthete [6]. Research into synaesthesia virtually vanished during the era of 20^th^ century Behaviourism, but was re-invigorated by the development of the ‘Test Of Genuineness’ (TOG) [7, 8] demonstrating synaesthesia has high consistency over time and re-opening the door to scientific enquiry into the phenomenon at multiple levels (sensory-perceptual, neuroimaging, epidemiological, and genetic). Eagleman and colleagues [9] adapted the TOG to an online standardised battery, the Synaesthesia Battery. The prevalence of synaesthesia is now estimated at 4.4% of the population [5], with a sex ratio of 6:1 female to male [10], although rates in females may be inflated [5]. Additionally, synaesthesia is familial [11]: Rich et al. [3] found that 36% of synaesthetes reported having at least one other family member who shared the condition. The neural cross-wiring theory of synaesthesia proposes this arises through hyper-connectivity between sensory brain regions as a consequence of a genetic predisposition towards reduced axonal pruning that typically results in cell death in non-synaesthetes [12–14]. This idea has received some support from neuroimaging [15] and genetic data [16].

Mirror-Touch (MT) synaesthesia is when a person reports experiencing touch in response to observing another person being touched [17]. It has a prevalence rate of 1.6%, based on a student sample rather than a general population sample [17]. Blakemore and colleagues [18] tested a single MT case seeing video clips of a human vs. an object being touched. Non-MT typical controls felt no response to seeing either human or object stimuli being touched, whilst the MT synaesthete ‘C’ rated a higher number of human clips as giving rise to sensation, and reported feeling nothing in response to objects. The authors interpreted this as showing that MT experience is specific to viewing a human being touched. Banissy and Ward [19] also reported that MT synaesthetes (*N* = 10) were significantly faster to name where a person was being touched on trials that were congruent with their synaesthetic experience of touch, and were significantly impaired in contrast to controls on incongruent trials. It is unclear if MT synaesthesia is specific to viewing a real person, rather than a dummy figure [20] or an object being touched [19] since in their Supplementary material Banissy and Ward [19] discuss some cases of MT synaesthesia also being induced by viewing objects being touched. Given the small samples that have been studied, the present study tests this specificity claim in a larger sample.

It is also claimed that MT synaesthesia leads to enhanced empathy [19] but again this was based on a small study of 10 MT synaesthetes, and although mean Empathy Quotient (EQ) [21] scores in the MT group did not differ from that of a control group, the MT group scored higher on one subscale on the EQ (emotional reactivity: mean = 17.2, standard deviation, SD = 1.41) compared to controls (mean = 13.56, SD = 0.46, *p* < 0.036). A study by Goller and colleagues found that individuals who report acquiring MT synaesthesia following amputation also had heightened emotional reactivity scores on the EQ [22], although this unusual form of MT synaesthesia (acquired, following surgery, and not developmental) means this is not strictly a replication of the initial Banissy and Ward study in 2007 [19]. It has also been reported that one MT synaesthete had increased mirror-neuron activity [18], based on a single case study. The synaesthete showed increased activity within SI, SII, and left premotor cortex, all associated with ‘mirror system activity’ [23]. Although from this early study it is unclear if this single case study is representative of MT synaesthetes as a group, the study by Holle and colleagues of 10 MT synaesthetes suggests it is [24]. Although MT synaesthesia was initially proposed to arise from greater activation of MT brain regions (normative somatosensory mirroring areas), other studies have suggested a different neural basis, namely atypical activation of self-other processing [17, 25–27]. The present study does not address these neural issues but simply tests the claim of enhanced empathy in the largest sample of MT synaesthetes yet collected, and across several empathy measures.

In addition, it has been claimed that individuals with autism spectrum conditions (ASC) may have reduced ‘mirror-neuron’ activity [28]. ASC has been linked with impairments in *cognitive empathy* [21]. This leads to the prediction that MT synaesthetes, if they have enhanced cognitive empathy, should be less likely to concurrently have an ASC. We also aimed to test this hypothesis in the present study. This is of particular interest because the opposite prediction is also plausible: that autism may be *more* common in synaesthesia if they both share reduced apoptosis, a neural mechanism that has been proposed for each condition separately [12–14, 29, 30]. Indeed, one interesting novel suggestion might be that autism in females may manifest as conditions like synaesthesia, where an obsessive focus on sensory sensitivity (including hyper-sensitivity) is recognized to be diagnostic in DSM-5 [29, 30].

In sum, the present study aimed to test four important claims/predictions that have been made about MT synaesthetes: that (1) they have enhanced empathy; (2) therefore that MT synaesthetes should be less likely than general population individuals without MT synaesthesia to have an ASC; (3) MT synaesthetes should have fewer autistic traits; and (4) that MT synaesthetes should only feel the perception of touch in response to viewing a human being touched.

## Method

### Participants

Participants were invited to take part via two online websites at www.cambridgepsychology.com and www.autismresearchcentre.com hosted by the University of Cambridge. In order to maximise the potential numbers in the MT and grapheme-colour (GC) groups, we also posted an advert at the following websites: the UK Synaesthesia Association (www.uksynaesthesia.com), the American Synaesthesia Association (www.synesthesia.info/), Synaesthesia Down Under (www.synesthesia.com.au/wp) and the Synesthesia List (www.daysyn.com/Synesthesia-List.html). In addition, synaesthesia researchers were sent the advert to forward to their synaesthesia participants (Anina Rich—Macquarie University; David Brang—University of California; Donielle Johnson—University of Cambridge; Jamie Ward—University of Sussex; Julia Simner—University of Edinburgh).

The study adopted a conservative approach to eligibility to ensure individuals in the synaesthesia groups were genuine cases of ‘developmental synaesthesia’, as opposed to ‘drug induced synaesthesia’ [29]. The Synaesthesia Screening Questionnaire (SSQ) was adapted from previous MT research [17] to define the groups and establish medical history. Synaesthetes with a history of hallucinogenic drug use were excluded from the study. Additionally, participants in the typical (non-synaesthete) control group with a recreational history of drug use were excluded. This was to tease apart MT synaesthesia from drug-induced altered mental experiences. Participants with a history of head trauma or epilepsy, brain tumours, stroke and migraine with aura, or severe comorbid psychiatric conditions, such as schizophrenia, bipolar disorder, or personality disorders, were also excluded, again to tease apart developmental MT synaesthesia from MT synaesthesia following acquired brain injury or perhaps misdiagnosed as part of a serious psychiatric condition. Those with less severe comorbid conditions, such as depression or anxiety, were included. In addition, individuals in the GC synaesthesia group were instructed to complete a validation test using the Synaesthesia Battery [9].

**Fig 1:**
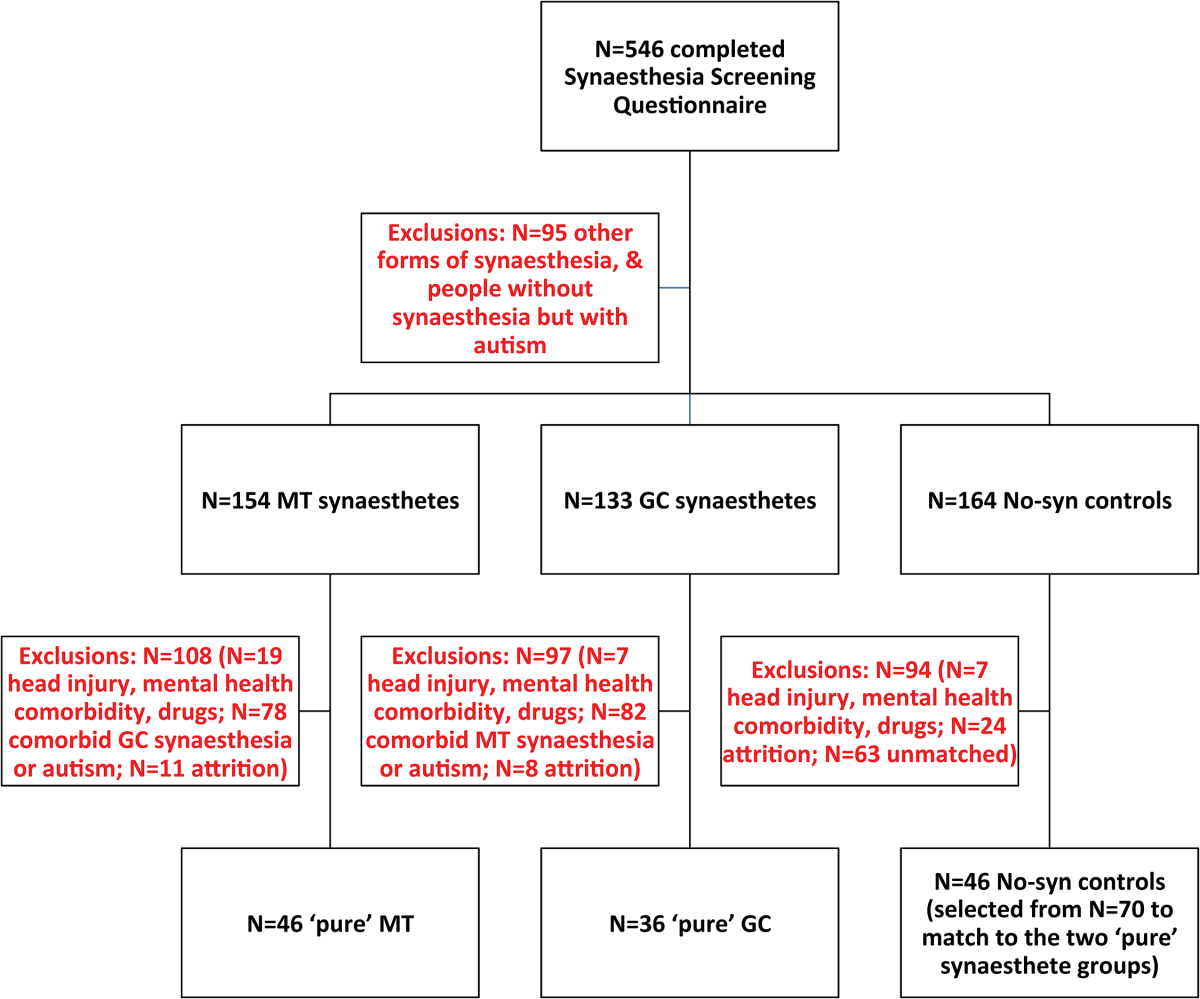
Flow chart of participant inclusion.

All participants were aged 16 years or older, and were recruited from English speaking countries. The initial cohort (*N* = 546) comprised a self-reported MT sample (*N* = 154), a self-reported GC sample (*N* = 133), and a no synaesthesia typical group (*N* = 164); *N* = 95 individuals were excluded on the basis of having forms of synaesthesia other than GC or MT and individuals with autism who did not have any form of synaesthesia. Groups were then refined on the basis of medical history exclusions, co-morbid diagnosis, and participant attrition (see Fig 1). This left three ‘pure’ groups: a pure self-reported MT group (*N* = 46), a pure self-reported GC group (*N* = 36), and a matched typical No-syn control group (*N* = 46). Demographic information for each group is displayed in Table 1.

**Table 1:** Age and Sex Distribution by Group.

Group	*N*	% Female	Mean Age (standard deviation, Range)
**MT SYN**	46	76.09	41.85 (11.82, 20–76)
**GC SYN**	36	77.78	41.44 (15.77, 16–81)
**No SYN**	46	78.26	44.20 (12.08, 24–70)

### Ethical Approval

**Fig 2:**
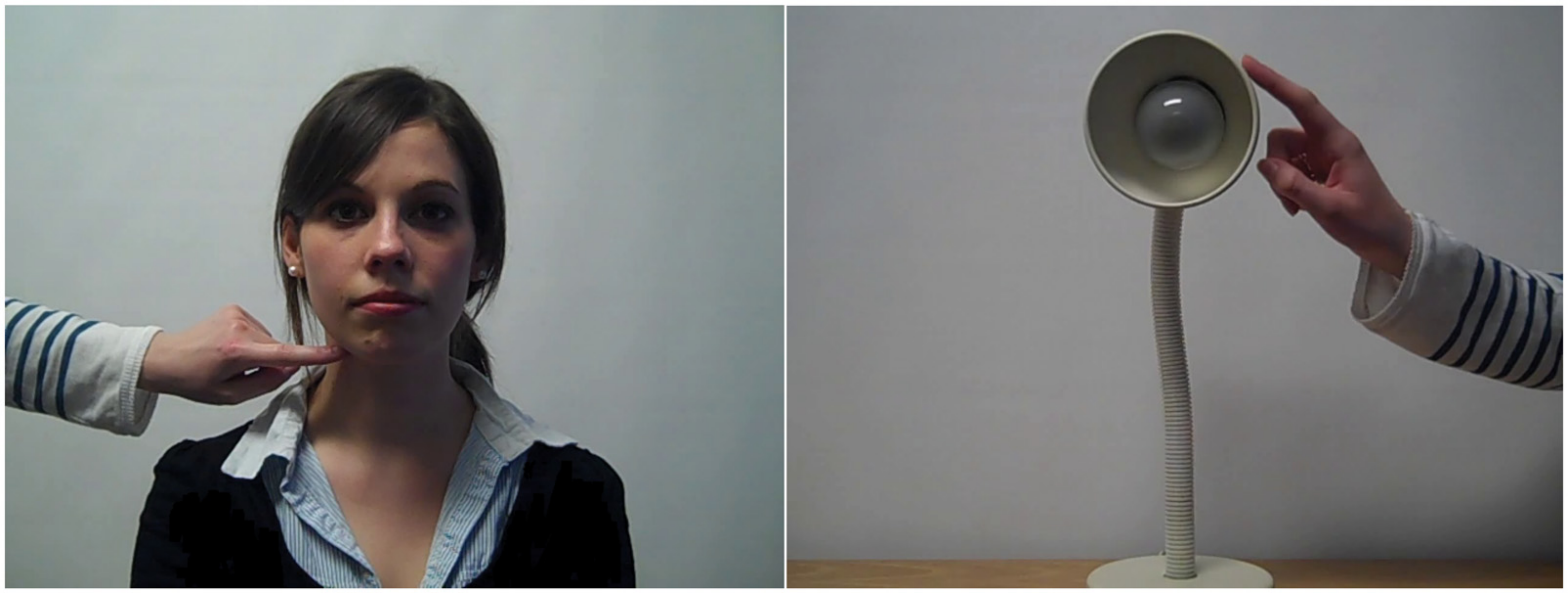
Snapshot of the Video Clips MT Validation Task. A. Human stimulus, Right Neck. B. Object Stimulus, Left Face.

Ethical approval was granted by the Psychology Research Ethics Committee, University of Cambridge, and consent was provided by the participants themselves in writing, online. The individual shown in Fig 2 is one of the co-authors (ER) who provided written informed consent (as outlined in the PLoS consent form) to have her face used in this photograph.

### Instruments

Two synaesthesia instruments were used: (1) *The Synaesthesia Screening Questionnaire (SSQ)* (online, adapted from Banissy et al. [17]), which included an MT synaesthesia item (“Do you experience touch sensations on your own body when you see another person's body being touched?”), and screened for medical history. (2) *The Synaesthesia Battery*. Participants in the GC group were asked to take the online Synaesthesia Battery [9] at www.synesthete.org/ and take two tests: (a) The GC consistency test, where participants were validated as genuine cases of GC synaesthesia if they scored between 0–1. The second task was the speeded congruency test, where the threshold for validation was 85–100% accuracy.

Three measures related to empathy were used: (1) *The Empathy Quotient (EQ*) [21] contains 40 items relating to empathy, with a maximum score of 80 and a minimum score of 0. Typical adults have a mean score of 42.1 (SD = 10.6). The EQ provides both measures of cognitive and affective empathy, as well as a measure of social skills, through 3 individual subscales identified by Lawrence et al. [31]. (2) *The ‘Reading the Mind in the Eyes’ Test (Eyes test)*. The Eyes test [32] asks participants to view 36 black and white photographs depicting the eye region of the face. They are asked to choose which word best describes what the person in the photo is thinking or feeling, from a choice of four complex mental state descriptive words. (3) *The Karolinska Directed Emotional Faces (KDEF)* [33]. An online version of this task was used [34], with 20 different colour photographs of faces depicting each of the 6 basic emotions plus 1 ‘neutral’ face, all taken from the same angle, with 10 actors shown twice for each emotion. Participants were asked to choose from 7 emotion words to describe each picture, and their response time for correct answers was measured. All 3 measures have been replicated independently suggesting they have excellent reliability and validity [21, 31–33].

Diagnosis of ASC was gathered at the point of registration on the two websites. If the person reported such a diagnosis, additional questions asked about specific subgroup diagnosis, and where and by whom the diagnosis was made. Only diagnoses that used DSM-IV or ICD-10 criteria (for Pervasive Developmental Disorder) and were made by a clinical psychologist or psychiatrist at a recognized clinic were included. For a measure of autistic traits, we used the *Autism Spectrum Quotient (AQ)* [35] that has a maximum score of 50 and a minimum of 0. The AQ has been extensively validated, and typical adults score on average 16.94 (95% CI 11.6, 20.0) [36].

MT synaesthetes further performed the *Video Clips MT Validation Task*. They were asked to complete this short task by Blakemore et al. [18] that looked at their perception of touch in response to viewing video clips showing humans and objects being touched. Participants were sent a DVD with 32 video clips, each lasting 4.5 seconds. Half the video clips depicted three different human actors being touched on their neck or face, either to the left or right hand side, by another person. The other half depicted a similar scenario with three different objects: a fan, a lamp and a speaker. The video clips were all made using the same camera and setting to minimise any bias. Additionally, they were all taken from the same distance and angle to reduce any extraneous variables. The clips were randomly ordered and then counterbalanced, to create a second version, to avoid order effects. Participants then randomly received either version, with a Touch Perception Questionnaire relating to the clips. This asked them to rate the intensity of tactile stimulation they felt on their own face or neck as a result of watching each clip on a 5-point scale, with 0 representing ‘no perceived tactile sensation’ and 5 signifying ‘very intense tactile sensation’. In addition to this, participants had to state the area and side of their body they felt the perception of touch, which was used to maintain their attention on each clip. See Fig 2.

### Procedure

Participants completed all tasks online at www.cambridgepsychology.com or, for participants with ASC, at www.autismresearchcentre.com. Participants in the self-reported GC synaesthesia group also visited www.synesthete.org to complete an online validation of GC synaesthesia. Participants in the self-reported MT group were sent the Video Clips Task, which included a DVD disc containing the video files and a paper Touch Perception Questionnaire (as described above) and returned it in a freepost envelope.

## Results

The distributions for each measure were checked for normality through measuring the skewness and kurtosis, frequency distribution, and the Kolmogorov-Smirnov test. The Eyes test and KDEF task scores were not normally distributed so non-parametric tests were employed. All other measures were normally distributed and were analysed with parametric tests. 47.2% of the GC synaesthete control group completed the Synaesthesia Battery (*N* = 17/36). They all passed the relevant thresholds (0–1 on the grapheme-colour consistency test / 85–100% on the speeded congruency test) for synaesthesic experiences. On this basis, it was assumed that the remaining *N* = 19 participants would have been validated, had they completed the Synaesthesia Battery, so all were considered validated and therefore *N* = 36 were included. The three ‘pure’ groups were age- and sex-matched; see Table 1.

**Fig 3:**
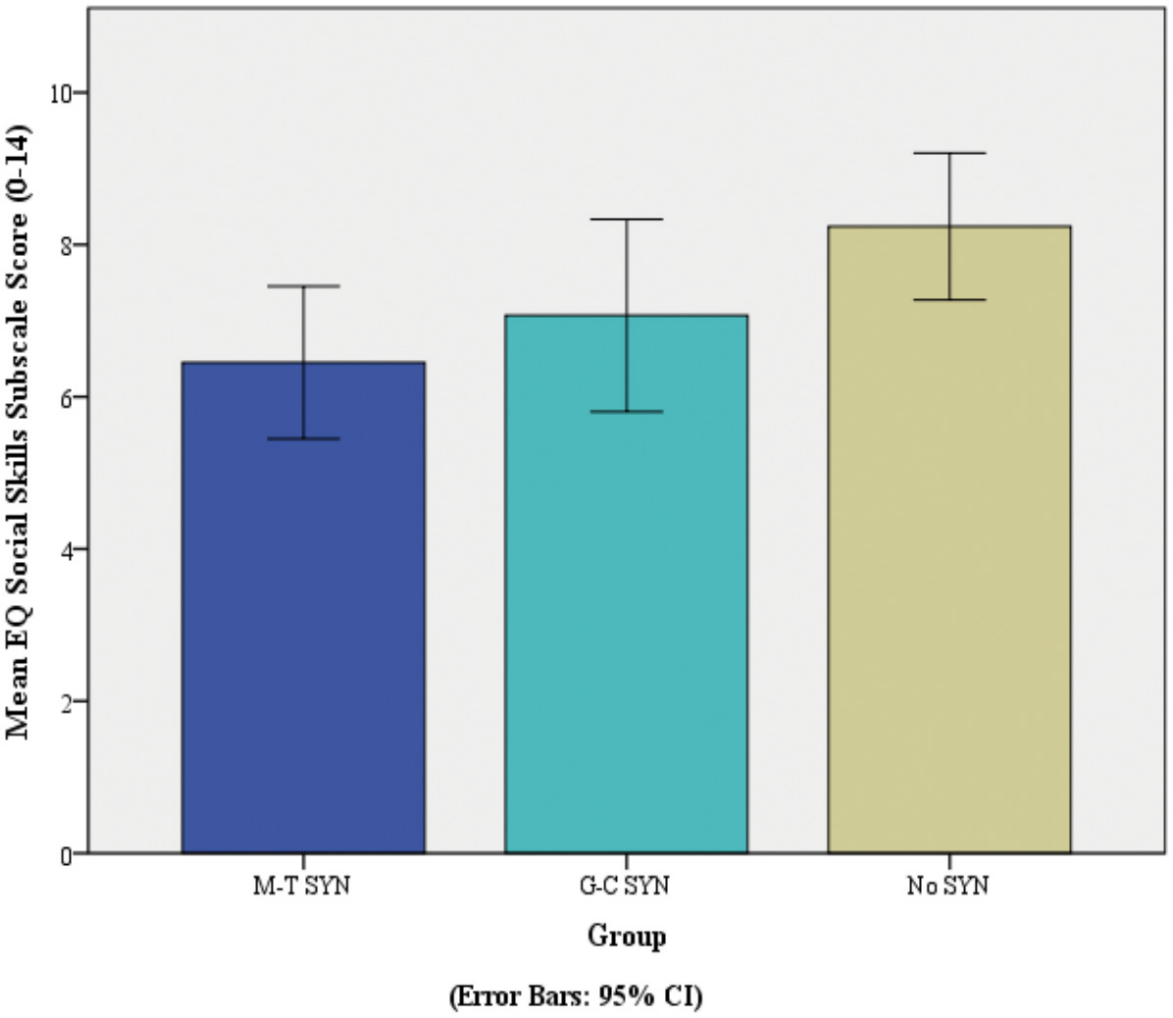
Mean EQ Social Skills Sub-score for individuals in the MT SYN Group, GC SYN Group, and No SYN Group * *p* < 0.05.

A one-way ANOVA was carried out to compare total EQ score; there was no significant difference between groups (*F* (2, 113) = 1.9, *p* = .16). Further analyses tested differences between groups on each of the three subscales of the EQ (Cognitive Empathy [CE], Emotional Responsivity [ER], and Social Skills [SS]). A one-way ANOVA revealed that there was no significant group difference in CE scores, *F* (2, 112) = .18, *p* = .83, or ER scores, *F* (2, 112) = 1.98, *p* = .14. The analysis did reveal a significant difference in SS scores between groups, *F* (2, 112) = 3.4, *p* = .037. See Table 2 and Fig 3. The effect size, calculated using eta squared, was .06, indicating a medium effect of group on SS scores. Post-hoc comparisons revealed that the mean SS score for the MT synaesthesia group (*mean* = 6.45, *SD* = 3.14) was significantly (*p* = .035, Bonferroni corrected) lower than the mean SS score for the typical control group (*mean* = 8.24, *SD* = 3.25). The GC group (*mean* = 7.07, *SD* = 3.33) did not differ significantly from either the MT or the typical control groups.

**Table 2:** EQ and Subscales: Cognitive Empathy (CE), Emotional Reactivity (ER) and Social Skills (SS) scores by Group.

	EQ total			CE			ER			SS		
Group	*N*	Mean	*SD*	*N*	Mean	*SD*	*N*	Mean	*SD*	*N*	Mean	*SD*
**MT SYN**	40	43.33	16.06	40	15.10	7.58	40	12.55	5.33	40	6.45	3.14
**GC SYN**	30	40.50	18.16	29	15.45	8.42	29	11.41	6.17	29	7.07	3.33
**No SYN**	46	47.67	14.9	46	16.04	6.26	46	13.98	5.33	46	8.24	3.25

On the KDEF task a Kruskal-Wallis test revealed that there were no statistically significant differences in accuracy (correct score) between the groups for any of the emotions (Angry: *H*(2) = 2.48, *p* = .29, Fearful: *H*(2) = .48, *p* = .79, Disgusted: *H*(2) = 4.79, *p* = .09, Happy: *H*(2) = 2.56, *p* = .28, Sad: *H*(2) = .83, *p* = .66, Surprised: *H*(2) = .75, *p* = .69, and Neutral: *H*(2) = .15, *p* = .93); See Table 3.

**Table 3:** KDEF Correct Scores for Angry, Fearful, Disgusted, Happy, Sad, Surprised and Neutral faces by Group.

	Emotion													
	Angry		Fearful		Disgusted		Happy		Sad		Surprised		Neutral	
Group	Mean / Median	*SD / IQR*	Mean / Median	*SD / IQR*	Mean / Median	*SD / IQR*	Mean / Median	*SD / IQR*	Mean / Median	*SD / IQR*	Mean / Median	*SD / IQR*	Mean / Median	*SD / IQR*
**MT SYN *N* = 25**	18.84 / 19	1.62 / 1.0	11.12 / 12	4.74 / 7.5	17.4 / 18	2.35 / 3.0	19.60 / 20	1.26 / 0	18.08 / 18	1.8 / 2.5	18.96 / 19	1.54 / 1.0	18.76 / 20	1.9 / 2.0
**GC SYN *N* = 14**	19.14 / 19.5	1.17 / 1.3	12.07 / 13.5	5.00 / 9.3	18.71 / 19.5	2.02 / 2.0	20 / 20	0 / 0	18.29 / 19	1.14 / 2.0	18.71 / 19.5	1.86 / 2.3	18.79 / 20	1.93 / 2.3
**No SYN *N* = 29**	19.38 / 20	0.82 / 1.0	11.72 / 12	3.97 / 7.5	17.83 / 19	3.32 / 2.5	19.90 / 20	0.31 / 0	18.14 / 19	2.55 / 3.0	19.24 / 20	1.09 / 1.5	19.07 / 19	1.1 / 1.0

SD: standard deviation; IQR: interquartile range. **Note:** Each target emotion has 20 items, with a maximum correct response rate of 20, and a minimum of 0.

In addition, the response time recorded for correctly identified target emotions (accuracy-adjusted response time) was analysed between the groups, controlling for a potential speed-accuracy trade-off [37]. Mean accuracy-adjusted response time was calculated by dividing the mean response time for each target emotion by the fraction of correct responses. A Kruskal-Wallis test revealed no significant differences by group in accuracy-adjusted response time for all emotions (Angry: *H*(2) = .34, *p* = .84, Fearful: *H*(2) = .24, *p* = .89, Disgusted: *H*(2) = 1.87, *p* = .39, Happy: *H*(2) = .77, *p* = .68, Sad: *H*(2) = .31, *p* = .86, Surprised: *H*(2) = .61, *p* = .74, and Neutral: *H*(2) = 1.34, *p* = .51); See Table 4.

**Table 4:** KDEF Accuracy-Adjusted Response Times (ms) for Angry, Fearful, Disgusted, Happy, Sad, Surprised and Neutral faces by Group.

	Emotion													
	Angry		Fearful		Disgusted		Happy		Sad		Surprised		Neutral	
Group	Mean / Median	*SD / IQR*	Mean / Median	*SD / IQR*	Mean / Median	*SD / IQR*	Mean / Median	*SD / IQR*	Mean / Median	*SD / IQR*	Mean / Median	*SD / IQR*	Mean / Median	*SD / IQR*
**MT SYN *N* = 25**	2431 / 2509	526 / 687	10540 / 5781	15640 / 5723	2649 / 2682	624 / 628	1861/ 1713	690 / 590	2617 / 2474	880 / 1118	2240 / 2095	666 / 574	2403 / 2120	733 / 883
**GC SYN *N* = 14**	2595 / 2589	706 / 1195	7773 / 4580	5059 / 10010	2461 / 2229	738 / 1074	1859 / 1727	477 / 735	2545 / 2453	890 / 1651	2502 / 2090	1200 / 1457	2306 / 1935	1017 / 1241
**No SYN *N* = 29**	2597 / 2425	849 / 1311	7122 / 6654	3764 / 4172	3041 / 2674	1704 / 738	1737 / 1623	503 / 414	2720 / 2388	1107 / 1564	2347 / 2324	710 / 981	2367 / 2166	739 / 607

SD: standard deviation; IQR: interquartile range

A Kruskal-Wallis test on the Eyes test performance revealed that there were no significant differences in correct scores between groups, *H*(2) = 1.46, *p* = .48; See Table 5. Finally, as Table 6 indicates, 30% (*N* = 41) of all self-reported MT synaesthetes (*N* = 135, from the originally recruited *N* = 154 MT excluding *N* = 19 with history of head injury, mental health comorbidity, and use of drugs) also reported having an ASC.

**Table 5:** The Eyes Test Correct Scores by Group.

Group	*N*	Mean / Median	*SD / IQR*
**MT SYN**	23	26.17 / 27.00	3.53 / 3.00
**GC SYN**	16	25.50 / 26.50	6.98 / 6.00
**No SYN**	33	27.18 / 28.00	3.16 / 5.00

SD: standard deviation; IQR: interquartile range

**Table 6:** Percentage of participants with MT synaesthesia who did, or did not, self-report having an ASC.

	Total		Male		Female	
Group	*N*	%	*N*	%	*N*	%
**MT with ASC**	41	30	16	12	25	19
**MT without ASC**	94	70	20	15	73	54
**Total**	135	100	36	27	98	73

**Note:** Total self-reported MT synaesthesia in the sample was *N* = 135, but one participant was excluded from the gender calculation due to lack of demographic information.

**Fig 4:**
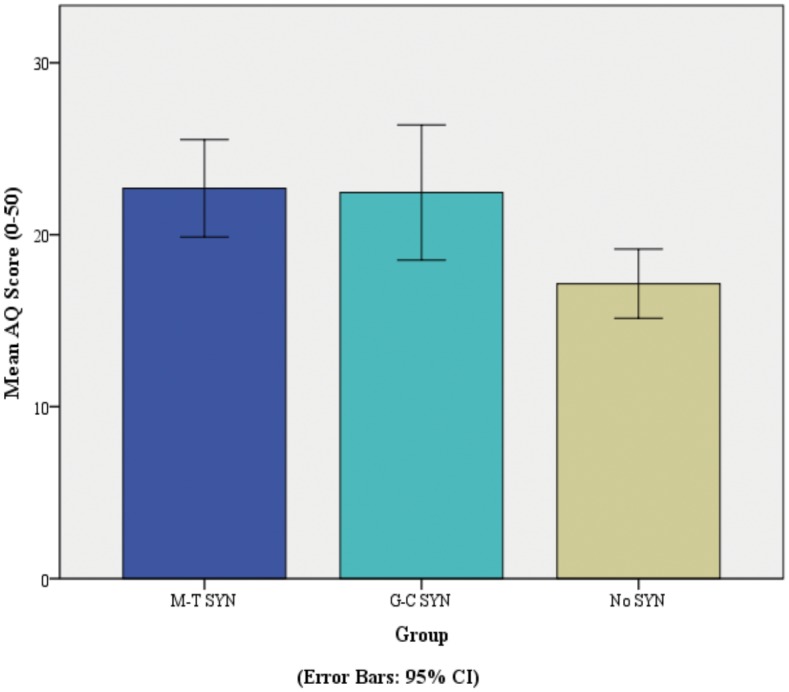
Mean AQ Score for the MT SYN Group, GC SYN Group and No SYN Group ** *p* < 0.01.

A one-way between-group ANOVA revealed a significant difference on the total AQ score between groups, *F* (2, 117) = 5.43, *p =* .006. The effect size, calculated using eta-squared, was .08, indicating a medium effect size of group on AQ. Post-hoc comparisons revealed that the mean AQ score for the MT synaesthesia group (*mean* = 22.7, *SD* = 9.21) was significantly (*p* = .011, Bonferroni corrected) higher than the mean score for the typical control group (*mean* = 17.15, *SD* = 6.77). Similarly, the mean AQ score for the GC synaesthesia group (*mean* = 22.45, *SD* = 10.72) was significantly (*p* = .032, Bonferroni corrected) greater than the mean score for the typical control group (*mean* = 17.15, *SD* = 6.77). There were no significant differences in the mean AQ score between the MT group and the GC group. See Fig 4.

**Fig 5:**
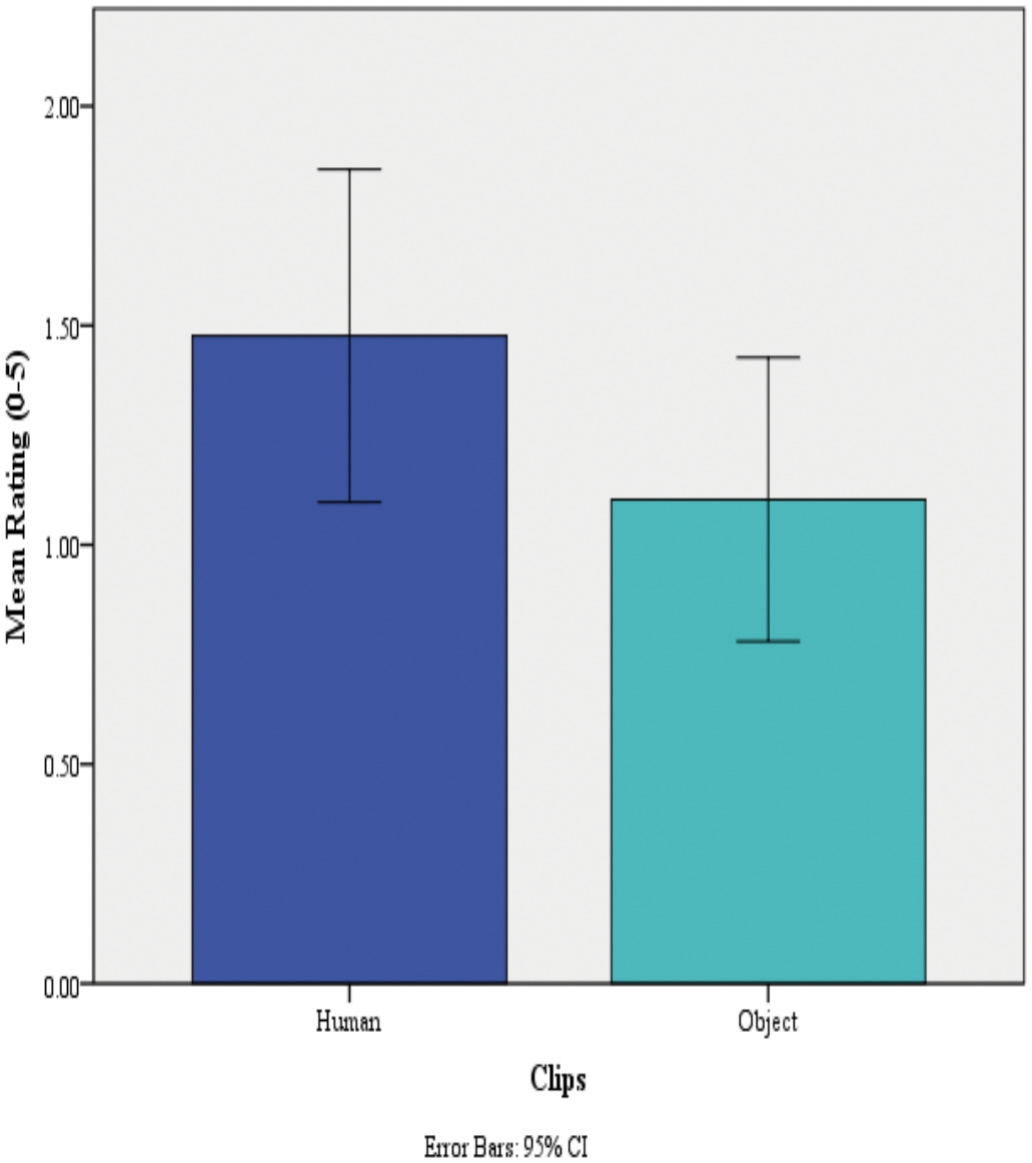
Mean Ratings for Human and Object Clips (from participants in the MT SYN Group) ** *p* < 0.01.

Finally, 44 participants in the total recruited MT group completed *The Video Clips MT Synaesthesia Validation Task*. A paired t-test was conducted to compare the mean ratings for human and object trials. There was a significant difference in scores, *t*(43) = 2.75, *p* = .009, which indicated that on average their felt tactile stimulation in response to humans were significantly greater than that in response to objects; see Table 7 and Fig 5. All MT synsaesthetes who completed this task reported MT experiences seeing another person being touched, which validates their synaesthesia. However viewing objects being touched did still trigger MT experiences (one-sample t-test [tested value = 0], *t*(43) = 6.87, *p* < .001).

**Table 7:** Mean Intensity of Tactile Stimulation Ratings (0–5) for Human and Object Clips, in the MT Group Participants.

Clips	*N*	Mean	*Standard deviation*
**Human**	44	1.48	1.25
**Object**	44	1.10	1.06

As a final note, because sex is linked to empathy and autistic traits [38–40] and that participant numbers completing different tasks differ (see Tables), sex ratios for each analysis (measure) were checked to ensure the groups were still matched. This revealed that the sex ratios were consistently matched across groups in all analyses/measures.

## Discussion

The present study tested whether self-reported MT synaesthetes (1) have enhanced empathy; (2) are less likely to have a concurrent diagnosis of ASC; (3) have fewer autistic traits; and (4) only experience MT sensations in response to viewing a person being touched. Contrary to predictions, MT synaesthetes scored in the average range on total EQ and did not score differently on the cognitive empathy or emotional reactivity sub-components of the EQ. There was a significant difference on the social skills subscale of the EQ, but in the opposite direction expected: the MT group scored significantly lower than the typical control group, suggesting that individuals with MT have a *reduced* aptitude for social situations. The GC group did not significantly differ from either the MT or the typical groups. On the Eyes test and KDEF measures, the groups also did not significantly differ from one another. This indicates that MT synaesthetes are no better, and no more automatic, at judging emotional states of others. This discrepancy with previous work could be because Banissy and Ward [19] only found a significant result on one subscale of one empathy measure, which could have been a Type I error, and because they only tested a small number of MT synaesthetes (*N* = 10), reducing the power to detect reliable results.

Also contrary to predictions, 30% of the initial sample of self-reported MT synaesthetes reported having an ASC, and there was a significant difference in AQ score between the ‘pure’ groups: The MT group and the GC group had significantly *higher* AQ scores than typical controls. Both of these findings suggest MT synaesthesia is not linked to enhanced cognitive empathy. Elevated AQ in synaesthesia is supported by other research: Baron-Cohen et al. [29] studied the savant ‘DT’ and found that as well as having synaesthesia he also met diagnostic criteria for Asperger Syndrome. A prevalence study [30] found the frequency of synaesthesia in individuals with ASC is 18.9%, almost three times greater than that in controls. This adds weight to the idea of a relationship between ASC and synaesthesia. It may even be that ASC is diagnosed more often in males because males show more obvious outward social difficulties whilst females with underlying but undiagnosed ASC more often succeed in masking or camouflaging their social difficulties [41], and are therefore more likely to be recognized and/or self-identify as having synaesthesia because the latter ‘diagnosis’ is based on atypical sensory functioning—a non-social core feature of ASC in DSM-5. That is, far from forms of synaesthesia (including MT synaesthesia) being less likely to co-occur with ASC, because of plausible shared underlying neural processes (such as reduced apoptosis), they may actually share many traits and co-occur more often than chance. This hypothesis (of elevated autistic traits or elevated rates of clinical ASC in females with synaesthesia) warrants further research.

Previous studies suggest that MT experiences are specific to viewing a human stimulus being touched [20]. The current study also found the mean rating for human clips was significantly higher than the mean rating for object clips, but the fact that viewing objects being touched did still trigger MT experiences in the MT group suggests that such experiences are not specific to viewing humans being touched.

The study has several limitations. First, this was an online/postal study that allowed us to increase numbers and to not be restricted to only recruiting participants from the UK. However, only 32.6% of the initially recruited MT group (*N* = 135, from the originally recruited *N* = 154 MT excluding *N* = 19 with history of head injury, mental health comorbidity, and use of drugs) completed the Video Clips MT Validation Task, which is a typical response rate for postal studies. Of the 44 MT synaesthetes who took the Video Clips Task, all of them reported MT synaesthesia experiences in response to viewing someone being touched, confirming their self-reports. Future replications could try to improve the response rate by in-person testing and by using other objective MT synaesthesia validation tasks [19]. There is no obvious reason to believe that those who completed the task were substantially different from those who did not. Second, we did not invite the GC synaesthete group or the non-synaesthete group to take the Video Clips Task, so we cannot be sure that none these individuals had MT synaesthesia. Future studies should test this in all groups but there is no reason to expect that those individuals who do not report having MT synaesthesia might have it. Third, only 47.2% of the GC synaesthesia group took the Synesthesia Battery because this involved leaving one website and going to another; this is a reasonable attrition rate but again there is no obvious reason to believe that those who completed the task were significantly different from those who did not, and all who completed the task passed it, which indicates that the synaesthetes were indeed real cases. Fourth, recruitment of participants was achieved from multiple sources, one of which was the Cambridge Autism Research Database (CARD), which may explain the high percentage of MT synaesthetes who reported a co-occurring diagnosis of ASC. Nevertheless, finding *any* co-morbid cases is against predictions from the enhanced empathy theory of MT, and the elevated rate of autistic traits in MT synaesthetes *without* ASC may imply that the higher AQ was driven by the subgroup of those who also had ASC.

## Conclusions

In conclusion, our results dispute the views that MT synaesthesia is linked with enhanced empathy, is less likely to occur with ASC or elevated autistic traits, and is specific to seeing a person being touched.
